# Long term survivors and immune biomarker analysis of Phase IIa, randomized study of GVAX pancreas and crs-207 immunotherapy in patients with metastatic pancreatic cancer

**DOI:** 10.1186/2051-1426-3-S2-P102

**Published:** 2015-11-04

**Authors:** Nitya Nair, Ed Lemmens, Amanda Enstrom, Shih-Yu Chen, Serena Chang, Dung Le, Elizabeth M Jaffee, Holden Maecker, Aimee L Murphy, Dirk G Brockstedt, Chan Whiting

**Affiliations:** 1 Aduro BioTech, Inc., Berkeley, CA, USA; 2 Stanford University, Stanford, CA, USA; 3 Johns Hopkins University, Baltimore, MD, USA; 4 The Sidney Kimmel Comprehensive Cancer Center at Johns Hopkins, Baltimore, MD, USA

## Background

Non-clinical and clinical combination studies with GVAX and live-attenuated, double-deleted (LADD) *Listeria monocytogenes* strains engineered to express tumor-associated antigens resulted in survival benefit and induction of cellular mediated immune responses.

## Methods

In a Phase IIa randomized study, patients with metastatic pancreatic ductal adenocarcinoma (PDA) treated sequentially with two vaccine-based immunotherapies demonstrated extended overall survival (OS) with tolerable side effects. Treatment included low-dose cyclophosphamide (CY) followed by GVAX pancreas, an irradiated GM-CSF-secreting allogeneic PDA cell vaccine, and subsequent administrations of CRS-207, a LADD vaccine engineered to express mesothelin. Study inclusion criteria were diagnosis of metastatic PDA, refusal or receipt of > 1 prior chemotherapy, ECOG 1 and adequate organ function. 90 patients were randomized 2:1 to receive 2 doses of CY/GVAX followed by 4 doses of CRS-207 (Arm A, median OS 6.1 months) or 6 doses of CY/GVAX (Arm B, median OS 3.9 months) (HR=0.54, one-sided p=0.011).

## Results

As of June 2015, 7 patients remain alive. Two patients continue on treatment: 1 original Arm A treatment (35.6 months on treatment); 1 Arm B on the 4^th^ course of rollover treatment (32 months on treatment). Five patients are currently in follow-up (Arm A) with a median time since first treatment of 36.1 months (range, 30.0 to 39.5 months). Peripheral blood mononuclear cells and serum were collected at baseline and over the course of treatment for immune monitoring and biomarker analysis. Here we report systematic cellular immunophenotyping and serum protein profiling in a sub-cohort of 38 PDA patients enrolled in this study. Mass cytometry and Luminex analysis of this sub-cohort revealed candidate biomarkers at baseline that were predictive of clinical outcome. Analysis of longitudinal time points using mass cytometry, flow cytometry, Luminex, Western blotting and ELISA are ongoing to identify mechanistic biomarkers that correlate with survival.

## Conclusions

These findings may inform future efforts to identify patients with productive responses that are most likely to benefit from immune-based therapy.

Clinical trial information NCT01417000

